# Enhancing structural health monitoring of fiber-reinforced polymer composites using piezoresistive Ti_3_C_2_T_x_ MXene fibers

**DOI:** 10.1038/s41598-024-78338-x

**Published:** 2025-01-19

**Authors:** Bircan Haspulat Taymaz, Handan Kamış, Michal Dziendzikowski, Kamil Kowalczyk, Krzysztof Dragan, Volkan Eskizeybek

**Affiliations:** 1 Department of Chemical Engineering, Faculty of Engineering and Natural Sciences, Konya Technical University, 42079 Konya, Turkey; 2 Airworthiness Division, Air Force Institute of Technology, 01-494 Warsaw, Poland; 3 Department of Materials Science and Engineering, Faculty of Engineering, Çanakkale Onsekiz Mart Universitesi, 17100 Çanakkale, Turkey

**Keywords:** Fiber-reinforced polymer composites, Structural health monitoring, Piezoresistive strain sensing, Ti_3_C_2_T_x_ MXene fibers, Composites, Electronic properties and materials

## Abstract

The anisotropic behavior of fiber-reinforced polymer composites, coupled with their susceptibility to various failure modes, poses challenges for their structural health monitoring (SHM) during service life. To address this, non-destructive testing techniques have been employed, but they often suffer from drawbacks such as high costs and suboptimal resolutions. Moreover, routine inspections fail to disclose incidents or failures occurring between successive assessments. As a result, there is a growing emphasis on SHM methods that enable continuous monitoring without grounding the aircraft. Our research focuses on advancing aerospace SHM through the utilization of piezoresistive MXene fibers. MXene, characterized by its 2D nanofiber architecture and exceptional properties, offers unique advantages for strain sensing applications. We successfully fabricate piezoresistive MXene fibers using wet spinning and integrate them into carbon fiber-reinforced epoxy laminates for in-situ strain sensing. Unlike previous studies focused on high strain levels, we adjust the strain levels to be comparable to those encountered in practical aerospace applications. Our results demonstrate remarkable sensitivity of MXene fibers within low strain ranges, with a maximum sensitivity of 0.9 at 0.13% strain. Additionally, MXene fibers exhibited high reliability for repetitive tensile deformations and low-velocity impact loading scenarios. This research contributes to the development of self-sensing composites, offering enhanced capabilities for early detection of damage and defects in aerospace structures, thereby improving safety and reducing maintenance expenses.

## Introduction

Fiber-reinforced polymer (FRP) composites play a pivotal role in modern structural engineering due to their high specific stiffness, particularly in aircraft primary structural components. However, FRP composites exhibit anisotropic behavior, and their tensile strength is much larger than the compressive and shearing strength. Consequently, they have been extensively applied for critical components and structures mainly subjected to tensile loadings^1^. However, FRP structural components are subjected to static and dynamic loads in different directions in flight and during maneuvers on the ground. Various failure modes, including fiber fracture, matrix cracking, fiber-matrix debonding, and delamination, arise during service life of composites. Identifying failure modes of composites during their service life poses challenges, as it often entails internal fiber breakage or delamination, which is difficult to detect with the naked eye^2,3^. Specifically, the mechanical properties of FRPs can be significantly affected by impact damage, causing a reduction in compressive strength of up to 60% compared to an undamaged laminate^4^.

Various non-destructive testing techniques, such as thermal wave imaging, laser vibrometry, X-rays, and acoustic emission, among others, have been employed to address and assess this challenge^5–7^. Nevertheless, these techniques exhibit drawbacks, encompassing high costs, suboptimal resolutions, and demanding time requirements^8,9^. Moreover, routine inspections, the conventional method of structural monitoring, need more capability to disclose incidents or failures that transpire between successive assessments. Consequently, there is considerable emphasis on conducting structural analyses on composite parts without grounding the aircraft^10^.

Structural health monitoring (SHM) is widely acknowledged as a promising approach to enhance safety by early detection and decrease maintenance expenses for composite aerospace structural components. SHM methods encompass both passive and active data acquisition systems. Various sensors, including acoustic^11^, piezoelectric^12,13^, fiber optic, laser vibrometers, and accelerometers^14^, are employed by both active and passive SHM systems to detect irregularities in composite structures.

Commercially feasible SHM solutions often require the installation of sensor systems after curing the polymer matrix, contributing to increased thickness and added weight. Moreover, the rising complexity and expenses of manufacturing, installation, maintenance, and data processing further compound the challenges. For example, one prominent technology in intelligent systems involves the integration of fiber-optic sensors within composite materials^15^, widely applied in various contexts, including safety-related components in aircraft. However, using fiber-optic sensors in embedded applications within composite structures can introduce elevated stress levels in resin-rich regions due to their larger diameters. Despite this drawback, they offer the benefits of a compact installation surface and immunity to electromagnetic interference^16^. Researchers have extensively explored these limitations in recent decades, resulting in the development of several SHM strategies. Among these, an auspicious approach for monitoring FRP composites involves piezoresistive strain sensing, wherein the electrical resistance of the sensor materials is observed^17,18^.

Advanced multi-functional composites endowed with integrated self-sensing capabilities have attracted significant interest from the aerospace and renewable energy industries^19^. These smart composites can constantly monitor their health state and offer real-time data on any changes in strain by incorporating strain-sensing devices in the composite structure^20^. This technology can transform the way of monitoring and maintaining composite structures by enabling early detection of damage or defects, allowing for timely intervention before catastrophic failures occur. The piezo resistivity in composite materials necessitates the incorporation of conductive materials, with the sensitivity of the sensing mechanism contingent upon the quality of filler dispersion, dimensions, conductivity, and geometries. The initial study on this topic was carried out by Schulte and Baron^21^, where they utilized the electrical resistance of carbon fibers with electrical conductivity to evaluate internal damage within carbon fiber-reinforced polymer (CFRP) composites. Their work included load and failure analyses of CFRP laminates, incorporating electrical resistivity measurements. Carbon nanomaterials such as carbon nanofibers (CNFs) and nanotubes (CNTs), as well as short and continuous carbon fibers (CFs) and carbon particles, can be used as these conductor components^22^. Nevertheless, previous endeavors faced challenges in fabrication and dispersion, potentially compromising the mechanical properties of composite structures^20^.

The distinctive 1D nanofiber architectures offer a straightforward design and can be configured into diverse shapes. Their flexibility, small size, and other inherent qualities make nanofibers well-suited for incorporation into FRPs, positioning them as excellent candidates for self-sensing composites. Alexopoulos et al.^23,24^ and Abot et al.^25^ showcased the utilization of CNT fibers for structural health monitoring in non-conductive glass fiber-reinforced polymer composites, mitigating challenges related to achieving uniform dispersions of carbon nanofillers. Khalid et al.^26^ employed piezoresistive carbon nanotube (CNT) fibers for structural health monitoring in composites with varied configurations. Park et al.^27^ integrated a CNT fiber sensor into a 1700 mm long right main wing, utilizing glass fiber-reinforced plastic skin. Strain measurements were performed during static loadings through the wireless SHM sensor node. However, numerous significant issues must be solved to promote future cutting-edge self-sensing composites. For instance, the sensitivity and capacitance performance of CNT fiber sensors and supercapacitors are often unsatisfactory, especially at low strains, limiting the application of CNT fibers as strain sensors in FRPs^28^.

The two-dimensional micrometer-scale layered structure of MXene offers distinct advantages, including high volumetric capacitance from rapid surface redox reactions, superior mechanical strength, and metallic conductivity, making it a leading choice among available nanomaterials for the development of advanced fibers^29^. Moreover, the hydrophilic surface groups (such as -F, -OH, and -O) on both sides of the nanosheets make MXene well-suited for solution processing. These attributes have facilitated successful preparations of MXene fibers using techniques such as wet-spinning, 3D printing, and biscrolling. Notably, during wet-spinning, the negatively charged MXene nanosheets autonomously assemble into a continuous fiber by restacking after extrusion from the MXene dispersion through a coagulation solution^13^. The strain-sensing performance and applications of MXene fibers represent an area of significant interest and potential impact in wearable electronics and structural health monitoring^30^. MXene fibers possess remarkable electrical conductivity and mechanical properties, making them promising candidates for strain-sensing applications. Integrating MXene fibers into flexible and lightweight sensor arrays makes it possible to accurately detect and monitor mechanical deformations and strains in real time^31^. These sensors can find applications in various fields, including wearable technology, human motion monitoring, structural health monitoring of infrastructure, and biomedical devices. With further research and development, MXene-based strain sensors can revolutionize how we monitor and interact with our environment, paving the way for advanced sensing solutions with broad societal impact^32^.

In this paper, piezoresistive MXene fibers were successfully fabricated using the wet spinning method, ensuring optimal morphological characteristics. Subsequently, the MXene fibers were attached to carbon fiber-reinforced epoxy laminates for in-situ strain sensing. In the literature, typically, the studies are focused on sensitivity characterization for high strain levels exceeding 1%^31,33,34^. In this paper, the strain level was adjusted to be comparable to levels encountered in practical applications, particularly in the aerospace industry. In^35^, the authors presented an example of a signal acquired by a strain gauge at an aircraft’s main landing gear during a full-stop landing. After aircraft deceleration, the strain level change compared to the aircraft in flight was about 0.16%. In another real case example of static load tests of unmanned aircraft vehicles^36^, the highest obtained strain levels for loads representing high-G maneuvers were below 0.1%. Therefore, the strain-sensing performance of MXene fibers was assessed through repetitive tensile tests at low strains (<0.15%) and hammer tap tests. The fibers were positioned perpendicular and parallel to the applied repetitive tensile load to scrutinize the influence of loading direction on the MXene fiber’s piezoelectrical performance. This research delved into the applicability of MXene fibers for self-sensing, revealing remarkable sensitivity in the strain range of 0 to 0.13%, with a maximum sensitivity of 0.9 at 0.13% strain. Also, the sensitivity of MXene fibers monotonically increased with the strain rate. Additionally, the MXene fibers exhibited high reliability for repetitive tensile deformations and low-velocity impact loading scenarios.

## Materials and methods

### Synthesis of Ti_3_C_2_T_x_ (MXene) aqueous dispersion

MXene sheets were synthesized using a modified minimally intensive layer delamination (MILD) method^37^. Initially, 1.6 g of lithium fluoride (LiF, 300 mesh, Sigma-Aldrich) was introduced into 40 mL of 12 M hydrochloric acid (HCl, 37%, Merck) and stirred at 300 rpm for 30 min to dissolve LiF. Subsequently, 1 g of Ti_3_AlC_2_ MAX powder (Nanografi, Turkiye) was slowly added while stirring at 1 g per 5 min. The etching reaction temperature was settled to 40 °C, and the etching solution was stirred at 300 rpm for 24 h. Next, the black solution obtained was washed via centrifugation for 5 min at 3000 rpm (Hermle Z206A). In this stage, at approximately pH 4, the black supernatant was collected in a separate beaker until a clear supernatant was obtained. Then, the accumulated black solution was concentrated with centrifugation for 60 min at 6000 rpm and washed with deionized water until the pH reached about 5.5. Delaminated 2D sheets of MXene were obtained, and samples were stored in the refrigerator. The desired MXene concentration was adjusted by diluting deionized water.

### Wet spinning of MXene fibers

The computer-controlled wet spinning unit was employed to produce MXene fibers (Supplementary Video)^38^. The MXene colloids (24 mg/ml) were injected via a blunt-tipped needle with 210 µm (27G) inner diameter at the rate of 0.8 ml/min into a rotating (20 rpm) aqueous chitosan (Mw = 600.000–800.000, Acros Organics) coagulation bath (1 wt%). The positively charged chitosan with long polymer chains triggers the polyelectrolyte complexation process in the coagulation stage^30^. Also, the positively charged chitosan coagulation bath demonstrated better mechanical strength and desired continuous characteristics^30,39,40^. The MXene colloid solidified in the coagulation bath, forming a continuous MXene fiber. After the MXene fiber was kept for 20 min in the coagulation bath, the fiber was extracted and rinsed with a wash bath of 7:3 water: ethanol bath for one hour, and the fibers were dried by hanging^33,41^.

### Composite laminate fabrication

Carbon fiber reinforced composites were manufactured from a unidirectional reinforced prepreg containing carbon fiber grade T700SC 12K and E722 epoxy resin supplied by Toray, Japan with a quasi-isotropic ([−45/90/45/0]3S) lay-up. The prepreg system is suitable for oven vacuum bag curing, press molding as well as autoclave curing. After laying down 24 layers on a stainless-steel sheet, the preform was subjected to a curing cycle in an autoclave at a pressure of 6.2 bar at 120 °C for 1 h. After curing, the specimen was machined to the final CAI dimension, which is 100x150x4 mm in accordance with ASTM D7137. Typical mechanical properties of specimens manufactured in accordance with the recommendations can be found in^42^.

### Materials characterization

The morphological features of MXene sheets were examined via transmission electron microscopy (TEM, JEOL JEM-1400 PLUS) and atomic force microscope (AFM, WITEC ALPHA 300 RA) images. An X-ray diffractometer fitted with a Cu K$\alpha$ radiation (40 kV, 30 mA) with an X-ray wavelength ($\lambda$) of 1.54 Å (XRD, Shimadzu XRD-6000) was utilized to investigate the structure of precursor and Ti_3_C_2_Tx MXene sheets. A digital microscope (Keyence VHX-6000) and an EDX-equipped scanning electron microscope (SEM, JEOL JSM-7100 F) were used to analyze the surface morphology and cross-section of MXene fibers. The electrical resistance of the different fibers was measured using bench digital multimeters (Fluke 233) with a custom-built two-probe unit with an interspacing of 12.25 mm and an analog impedance analyzer (Digilent, Analog Discovery 2). The initial resistance values of about 40 $\Omega$ were recorded before the tests.

### Material preparation for sensing measurements

**Fig. 1:**
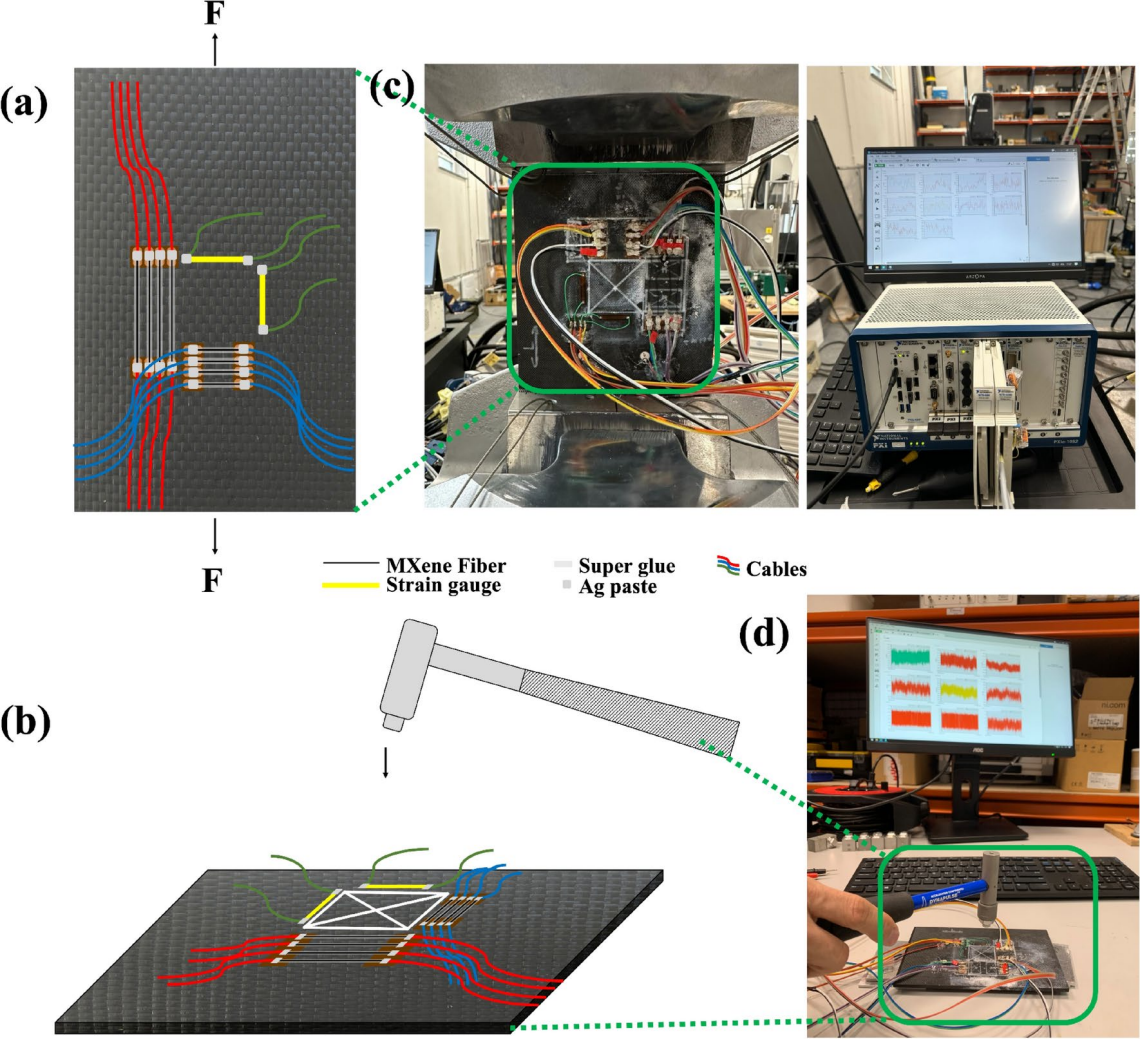
MXene fiber-attached fiber-reinforced composite samples for: (**a**) cyclic tensile (**b**) hammer tap impact tests and photographs taken during (**c**) cyclic tensile (**d**) hammer tap impact tests.

The current-voltage (I-V) characteristics of MXene fibers were determined using Keithley 2614B source measure unit (SMU). The fibers were subsequently connected in pairs to the device in a 4-wire sensing configuration, which positively affects the quality of the measurement results. The study was conducted in two runs for every fiber for DC excitation voltage of the fibers with a sweep range from −5 to +5 V with a step of 12 mV^43^. Following, a fiber-reinforced composite sample was prepared by adhering to two groups of MXene fibers with different lengths on the composite laminate (Fig. 1a, b). The first group contained four MXene fibers aligned along the longitudinal direction (FL1-4) with a length of 35 mm. Each fiber was located parallel to each other with a distance of 3 mm. The second group also contained four MXene fibers aligned along the transverse direction (FT1-4) with a length of 20 mm and a distance of 3 mm from each other. MXene fibers were kept straight and stretched while attaching to copper conductive ports using a conductive silver paste^44^. A non-conductive layer of superglue was applied to cover and fix the fibers on the composite laminate. For each direction, MXene fibers were transferred onto the copper ports with a 3 mm distance between each other. Before attaching copper ports to the composite sample, an epoxy film with a 35-micrometer thickness was applied on the composite laminate surface using epoxy adhesive to avoid the short circuit. The distance between the closest MXene nanofiber and the sample center was 12.5 mm for both directions.

To compare the strain measurement from the MXene fiber sensors, two 350 $\Omega$ strain gauges (CEA-06-125UN-350, Micro Measurements) with a grid resistance of 350 ± 0.3 $\Omega$ and gauge factor of 2.155 ± 0.5% at 24 °C were also attached on the composite laminate to measure longitudinal and transverse strain during cyclic tensile and tap tests. The strain gauges are applied on the same side of the MXene fiber sensors. The top layer of resin is sanded down to expose the fibers on the smooth side. The fiber dust was removed using a dry cloth. Conditioner A is then used to remove any contaminants on the surface. The neutralizer restores the pH to 7, counteracting the acidic nature of the conditioner. The surface was cleaned thoroughly to ensure proper adhesion of the gauge. The catalyst was then applied to the back of the strain gauge to increase the reaction rate. The strain gauge was adhered to the middle of the sample using M200 adhesive (Fig. 1a, b). The wires were then soldered onto the strain gauge. Once the wires were attached, the strain output was checked to validate the connections.

### Mechanical tests

The strain-sensing characteristics of the MXene fiber-attached fiber-reinforced composite samples were evaluated by cyclic tensile and hammer tap impact tests. The cyclic tensile tests were conducted on MTS Acumen 12 Electrodynamic Test Systems, a universal testing machine. The strain was measured using a strain gauge attached to the composite samples. To characterize the maximum electrical resistance, change rate, and gauge factor of the MXene fiber, a single loading cycle at 60 MPa at 3 Hz was applied in tension-tension mode, demonstrating high-G maneuvers of an unmanned aircraft^36^. The measurements during cyclic tensile tests were conducted using the NI PXI measurement platform with the autonomous controller PXI-8861 and NI FlexLogger software. The PXIe-4339 module, allowing for multi-channel acquisition of differential voltages, was utilized to measure MXene fibers’ resistances (Fig. 1c). To ensure measurement accuracy, six fibers from the specimen were incorporated into Wheatstone bridges with nominal arm resistances of 350 $\Omega$. The bridge circuits were balanced by serially connecting the fibers with resistors selected to provide the appropriate equivalent resistance. The circuits were powered by a constant voltage of 2.5 V. Additionally, using a supplementary PXIe-4300 module, signals from the load frame controller providing information about the current force and displacement of the actuator were simultaneously acquired. The cyclic tensile tests were conducted with the force value controlled, utilizing a stepwise value for static tests and a sinusoidal function with a frequency of 3 Hz for dynamic one, while acquiring signals at a constant frequency of 100 Hz each time.

### Hammer tap tests

**Fig. 2:**
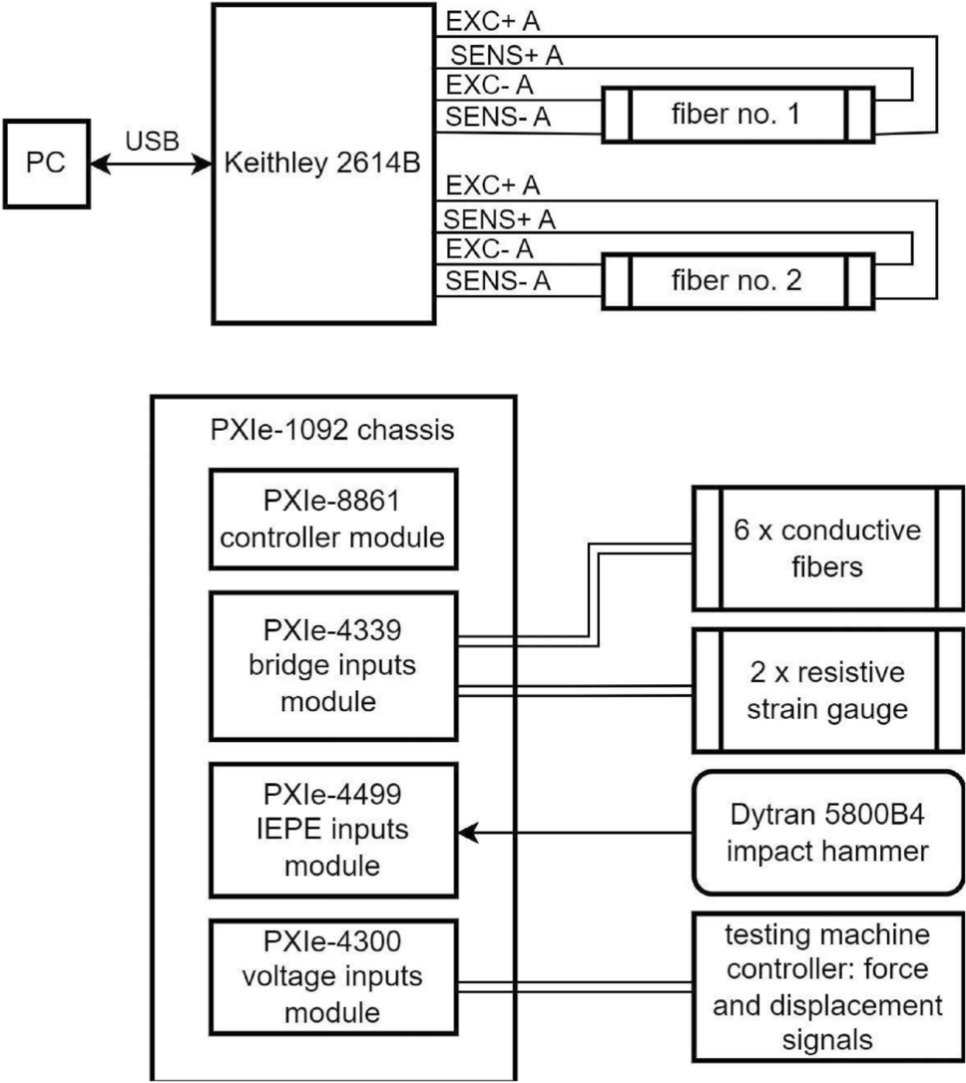
The diagram of the NI PXI acquisition system configuration.

Hammer tap tests were conducted to assess the piezoelectric behavior of MXene fiber sensors under low-velocity impact loading. An impact hammer (Dytran Dynapulse$^ {\text{TM}}$ model 5850B), equipped with a piezoelectric force sensor on the striking surface of the hammerhead, was utilized to apply impact loads to the composite laminate (Fig. 1b). The hammer had a rigid plastic tip with a short contact time and a wide frequency band of transmitted frequencies. At least 50 strokes were applied to the intersection of the diagonal of the square shown in Fig. 1, ranging from 100 to 400 N, and the impact loads were concurrently recorded throughout the tests. To measure the change in electrical resistance of MXene fibers, the measurement system used in the tensile tests was expanded with the PXie-4499 module to connect impact hammer (Fig. 1d). This setup enabled recording the impact force amplitude and its distribution over time during contact with the sample. During the impact tests, readings from the hammer, as well as from the electroconductive fibers and strain gauges, were captured at a consistent frequency of 25 kHz. The block diagram of a complete configuration of the measurement setup is presented below (Fig. 2).

## Results and discussion

### Preparation of MXene and MXene fiber

**Fig. 3:**
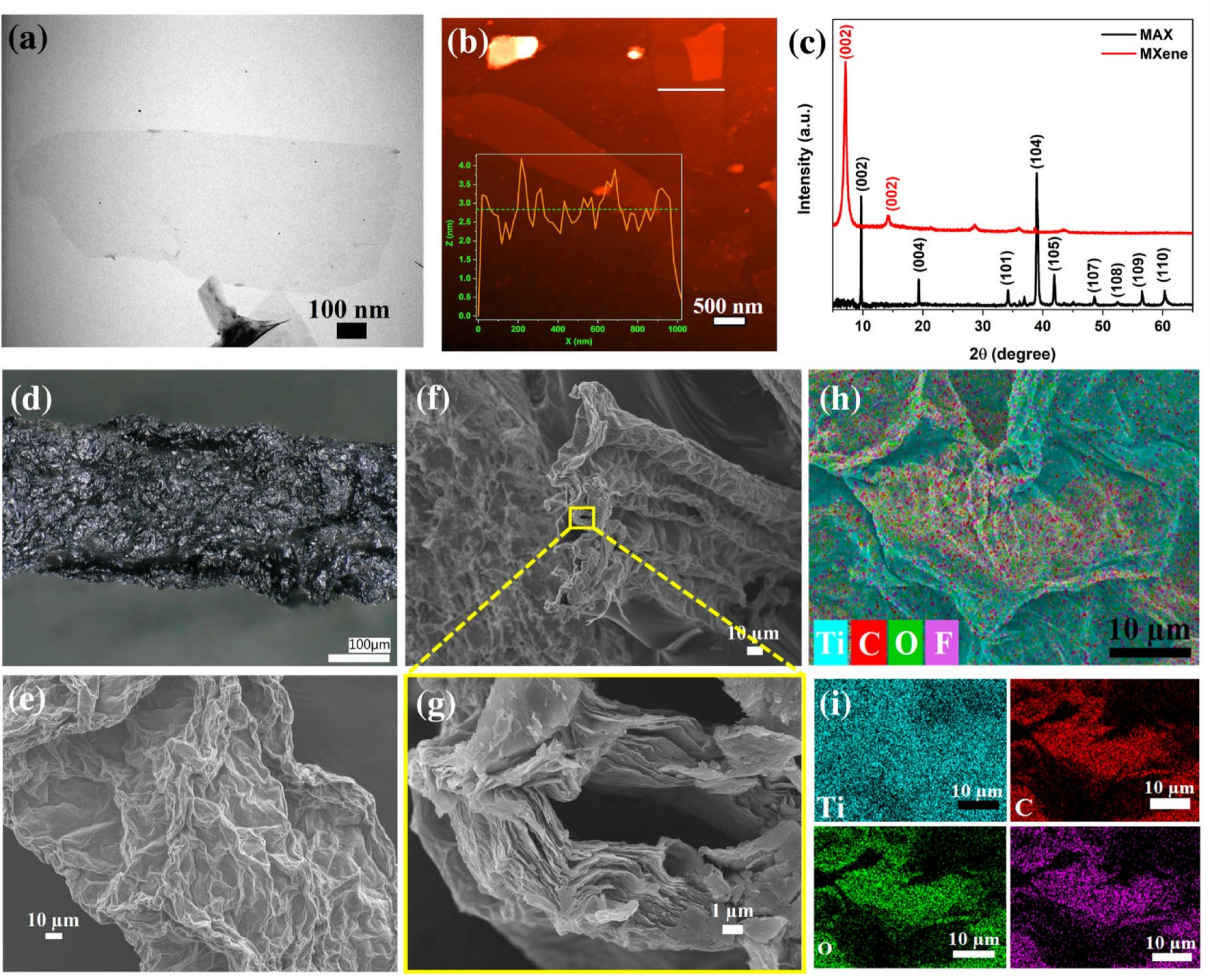
(**a**) TEM and (**b**) AFM image of MXene sheets and the inset graph shows the height profile of MXene sheets, (**c**) XRD patterns of precursor MAX and MXene, (**d**) optical microscope image of MXene fiber and SEM images of MXene fiber: (**e**) surface, (**f**) cross-section, and (**g**) enlarged cross-section in yellow area. (**h**) EDS elemental map of MXene fiber and mapping images of (**i**) Ti, C, O, and F.

The MXene was produced by selectively etching the Al layer from the $Ti_3AlC_2$ MAX phase with HCl and LiF. The morphological properties of MXene sheets were investigated via TEM and AFM images. The TEM image in Fig. 3a indicates MXene sheets represent a high transparency and prove that MAX was exfoliated to a few layered MXene. Moreover, the thickness of MXene sheets was determined via AFM images in Fig. 1b, and the height profile graph was given in inset Fig. 3b. The thickness of the MXene sheet is MXene about 2.8 nm, which is compatible with the definition of few-layered MXene^45^. To acquire more details about the structure of the samples, XRD was employed to examine the bulk MAX phase and delaminated MXene (Fig. 3c). After etching and delamination, the main (104) peak at $2\theta \approx 39^\circ$ of MAX (JCPDS No. 52-0875) is almost disappeared, displaying the Al-layers are selectively etched from the precursor^46^. Also, the presence of the characteristic (002) peak of MXene was observed at $2\theta \approx 7.1^\circ$, which is lower than that of the bulk MAX phase ($2\theta \approx 9.7^\circ$), demonstrating an enhancement of the interlayer spacing of MXene^45^. The wrinkled surface of the MXene fiber was observed in an optical microscope image (Fig. 3d). Also, the surface and cross-section morphological features of MXene fiber were deeply investigated via SEM analysis (Fig. 3e–g). The SEM images revealed the high alignment of MXene sheets along the fiber axis and the wrinkled surface of the MXene fiber (Fig. 3e). Moreover, the cross–section SEM images of MXene fiber prove the alignment of MXene sheets (Fig. 3f–g). EDS mapping image of the MXene fiber surface and with the corresponding Ti, C, F, and O mapping images are given in Fig. 3(h–i). The EDS mapping images demonstrate the formation of surface terminal groups and the uniform allocation of elements.

### Electrical properties of MXene fibers

**Fig. 4:**
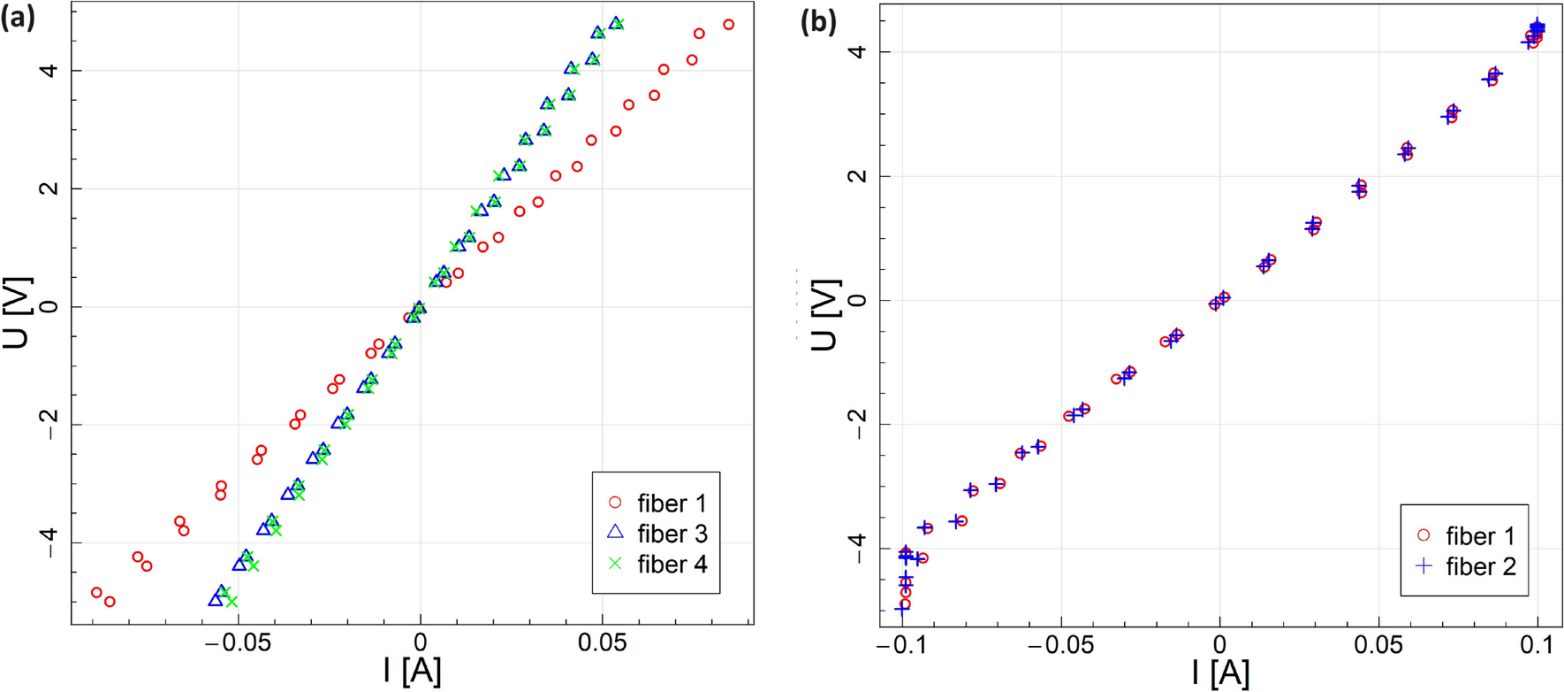
Current-voltage characteristics for (**a**) longitudinal and (**b**) transversal MXene fibers in the unloading state at room temperature.

The electrical properties and stabilities of the MXene fiber piezoresistive strain sensors were initially examined in the unloading state, where no strain or force was exerted, to assess their potential. Initially, the sensors were exposed to a range of voltages (−5 to 5 V), and the resulting current-voltage curves were plotted. Figure 4 shows that the produced sensors exhibited a linear and ohmic response, demonstrating that the sensors’ response endures consistently throughout a broad spectrum of voltages, a vital characteristic of a strain sensor. Linear models were fitted to the obtained data, and based on the model coefficients, the resistance of longitudinal and transverse MXene fibers and 95% confidence intervals for resistance values were obtained in load-free conditions at room temperature. For transversal fibers, the measurements were valid for only 2 fibers.

Based on our unloading state resistance tests, the MXene sensors demonstrated an initial resistance within the range of 50 to 92 ohms (Table 1). The observed variability in resistance values can be attributed to the morphology of the fibers and their wrinkle density, as elucidated by microscopic analyses conducted on MXene fibers. Furthermore, longitudinal MXene fiber sensors exhibited comparatively higher resistances in contrast to their transverse counterparts, owing to their extended lengths^47^.

**Table 1:** Initial resistance of MXene fibers.

	Code	Estimated R	Lower confidence bound	Upper confidence bound
Longitudinal	FL1	57.18	57.08	57.28
	FL3	90.37	90.23	90.52
	FL4	92.15	91.95	92.35
Transversal	FT1	42.60	42.50	42.70
	FT2	42.71	42.62	42.90

**Fig. 5:**
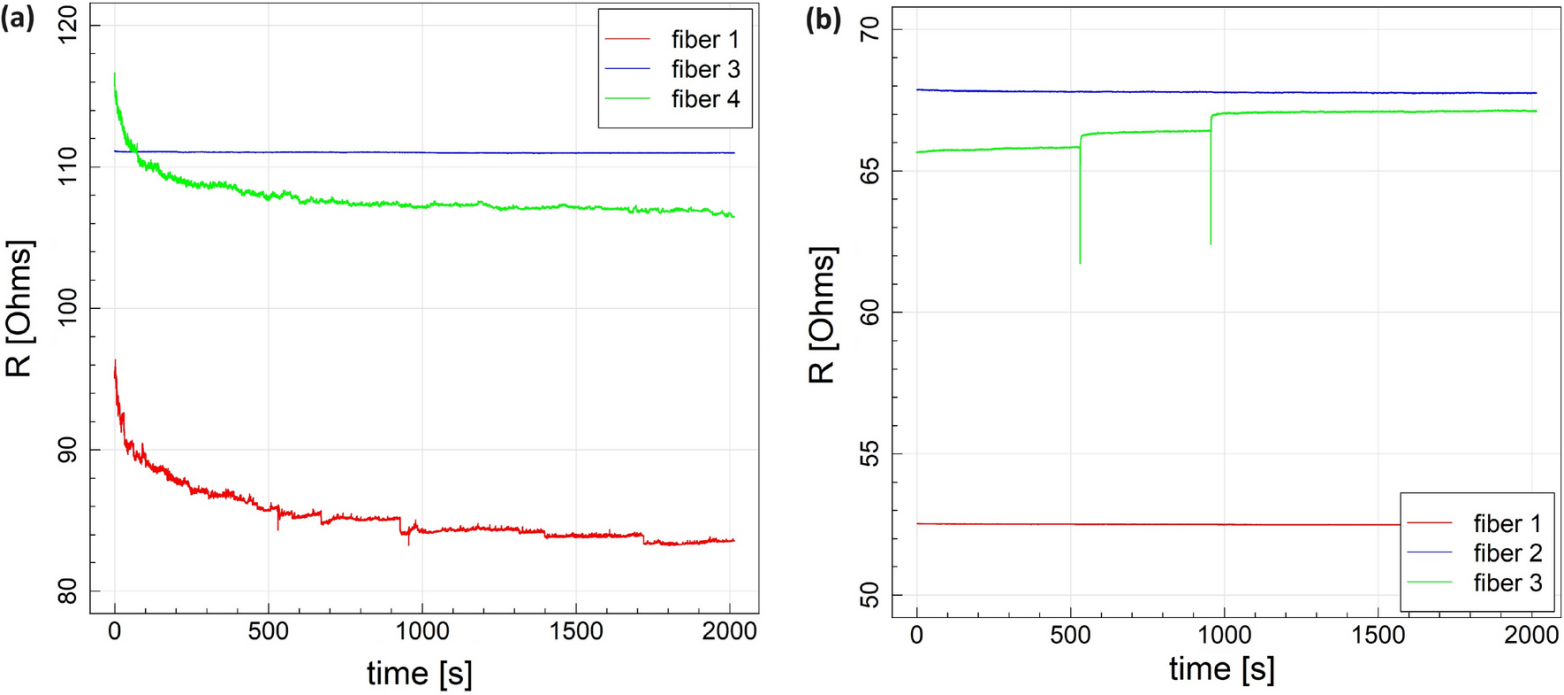
Resistance stability of MXene fibers in (**a**) longitudinal and (**b**) transverse direction at room temperature.

The stability of nanofiber strain sensors under constant voltage and temperature conditions refers to their ability to maintain consistent and reliable performance over extended periods^48^. This stability is crucial for ensuring the accuracy and longevity of the sensors^49^. Reliable and stable sensors provide accurate data over prolonged periods, enabling better decision-making, enhanced safety, and improved performance in diverse fields. The stability of sensors at the unloading state was assessed by subjecting MXene fibers to show how the resistance of the MXene fiber sensors changed over time at a continuous voltage of 5 V, as seen in Fig. 5. For the longitudinal fibers (Fig. 5a), FL3 swiftly attained stability without initial resistance fluctuations, akin to the immediate stability. Conversely, FL4 exhibited a slight decrease (8.6%) in resistance within the initial 500 seconds, followed by stabilization. Moreover, FL1 displayed a gradual decline in resistance (12.5%) over 1500 seconds before reaching a stable state. On the other hand, for transversal fibers, FT1 and FT2 represent no change in resistance at room temperature, while FT3 exhibits a slight increase in resistance (Fig. 5b). The sensing behavior of MXene fibers can differ due to a combination of factors related to material properties, fabrication techniques, structural characteristics, and environmental conditions. Our observations support that the change in morphology of MXene fibers, such as their diameter, aspect ratio, and wrinkles on the surface, influence their mechanical and electrical properties and, consequently, their sensitivity to strain.

### Electromechanical analysis

The mechanical characteristics of CFRP samples with MXene fibers were evaluated using cyclic tensile tests. As a material experiences tensile loading, it undergoes elongation (L increases) and reduction in cross-sectional area (A decreases), leading to a rise in resistance (R). The piezoresistive characteristics of MXene fibers were assessed within the early-elastic range of CFRP (with a maximum strain value of 0.13%) through cyclic tensile testing, aiming for relevance to practical scenarios, notably in the aerospace sector^35^.

The NI Compact Data Acquisition system was used to capture the variations in resistance over time for the MXene fiber sensors during the cyclic tensile test. The calculation of the resistance variation was performed using the following expression:

1 $$\begin{aligned} \frac{\Delta R}{R_0}\times 100 = \frac{R_i-R_0}{R_0}\times 100 \end{aligned}$$

where $R_i$ represents the resistance at a specific moment, and $R_0$ represents the initial resistance, measured in the unloading condition. The gauge factor *GF* and sensitivity of the sensors with respect to strain $\varepsilon$ were determined using the following definition:

2 $$\begin{aligned} GF = \frac{\Delta R}{R_0\varepsilon }. \end{aligned}$$

**Fig. 6:**
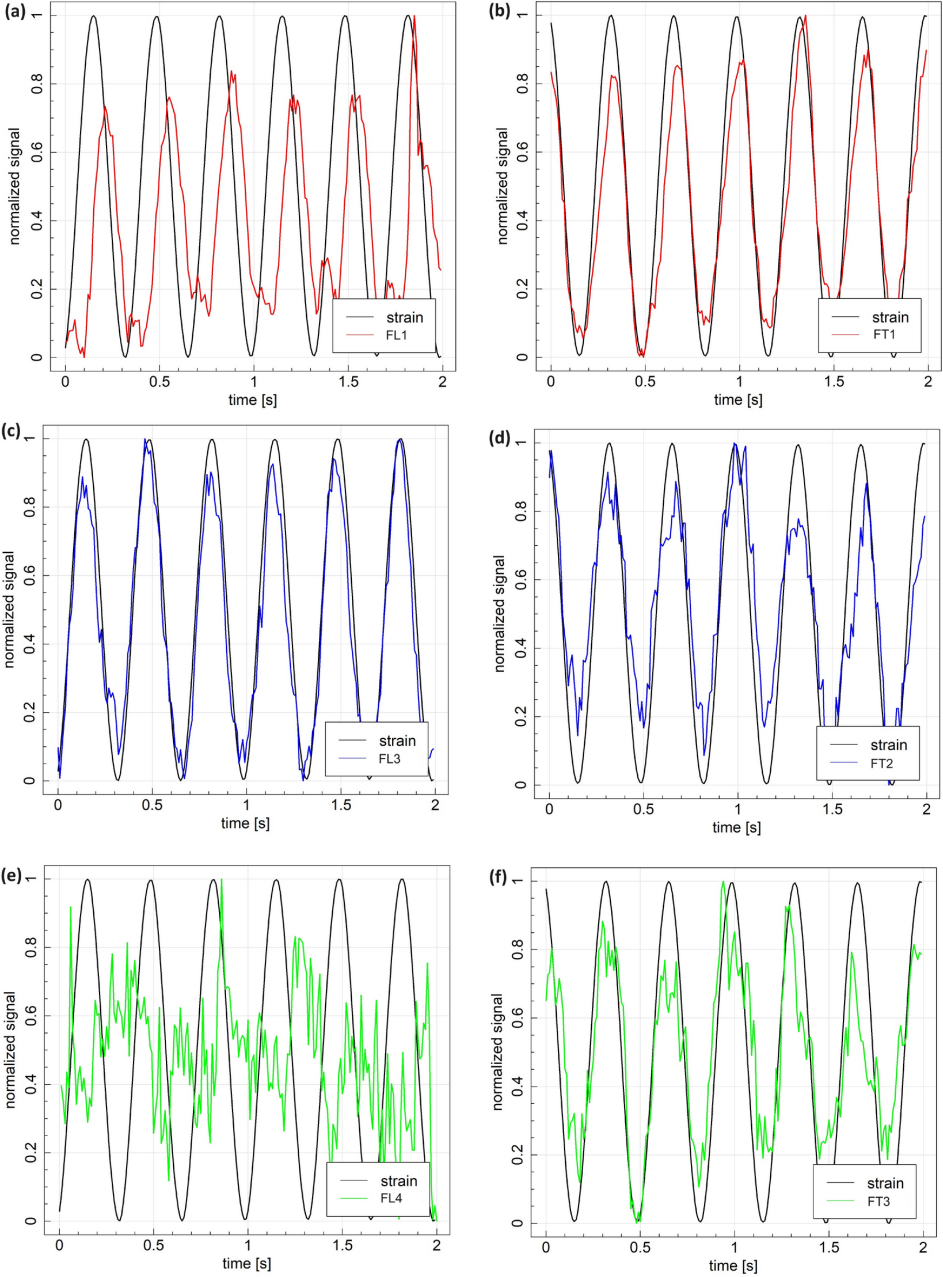
Example of normalized signals acquired during cyclic loading: (**a**, **c**, **e**) sensors aligned with the applied force direction and (**b**, **d**, **f**) sensors aligned in perpendicular direction.

The laminated composite specimen was subjected to cyclic tensile loading with a stress ratio of $R>0$ and maximum stress level at 60 MPa (up to 0.13% strain), i.e. in a purely linear elastic regime of the composite laminate . The strain gauge signal was also implemented in the figure for comparison. The MXene fiber sensors successfully captured and matched the elastic region of the composite, indicating that the adhesion of MXene fiber sensors on the surface of the composite laminate is feasible. The FL3 closely tracked the strain gauge signal, exhibiting a slight discrepancy in the rate of change of electrical resistance peaks (Fig. 6c). Applying cyclic tensile loading led to a change in piezoresistive characteristics in the MXene fibers, accounting for increased and decreased electrical resistance stemming from loading and unloading^24,50^. These piezoresistive characteristics result from changes in the dimensions of the MXene fibers, i.e., elongation in the longitudinal direction and shrinkage in the transverse direction under tensile loading^26,51^.

On the other hand, for FL1 (Fig. 6a), the electrical resistance change rate was not distinctive and exhibited fluctuations. This can be due to the thick layer of adhesive used for fiber integration with the host structure, which may result in inconsistent adhesion between the MXene fiber sensor and the carbon fiber-reinforced epoxy composite laminate (CFRP). Accordingly, the inconsistent adhesion can lead to disparities in the electrical resistance change rate and its peak. In addition, the imperfections in the connection interface between the fiber and the wire used to connect to the measuring device may also lead to disparities in the electrical resistance change rate and its peak^43^. FL4 exhibited an incomplete noisy signal with a poor resolution (Fig. 6e). MXene fiber sensors aligned to the transverse direction (perpendicular to the applied load) were in agreement with the strain in that direction (Fig. 6b, d, f). Note that the strain level in this direction due to the applied load was significantly lower than in the longitudinal direction. The variation in sensing abilities observed among the MXene nanofibers can be attributed to non-uniform epoxy coating or contact resistance. When fibers on the composite laminate are fixed, the application of silver conductive adhesive and epoxy coating may not be uniform across all fibers. Variations in coating thickness or coverage could lead to differences in the electrical contact and isolation of the fibers, affecting their sensing abilities. Besides, the quality of electrical contact between the fibers and the conductive adhesive can vary, resulting in differences in contact resistance. Poor contact can lead to higher resistance and reduced sensitivity in some fibers compared to others^52^.

**Fig. 7:**
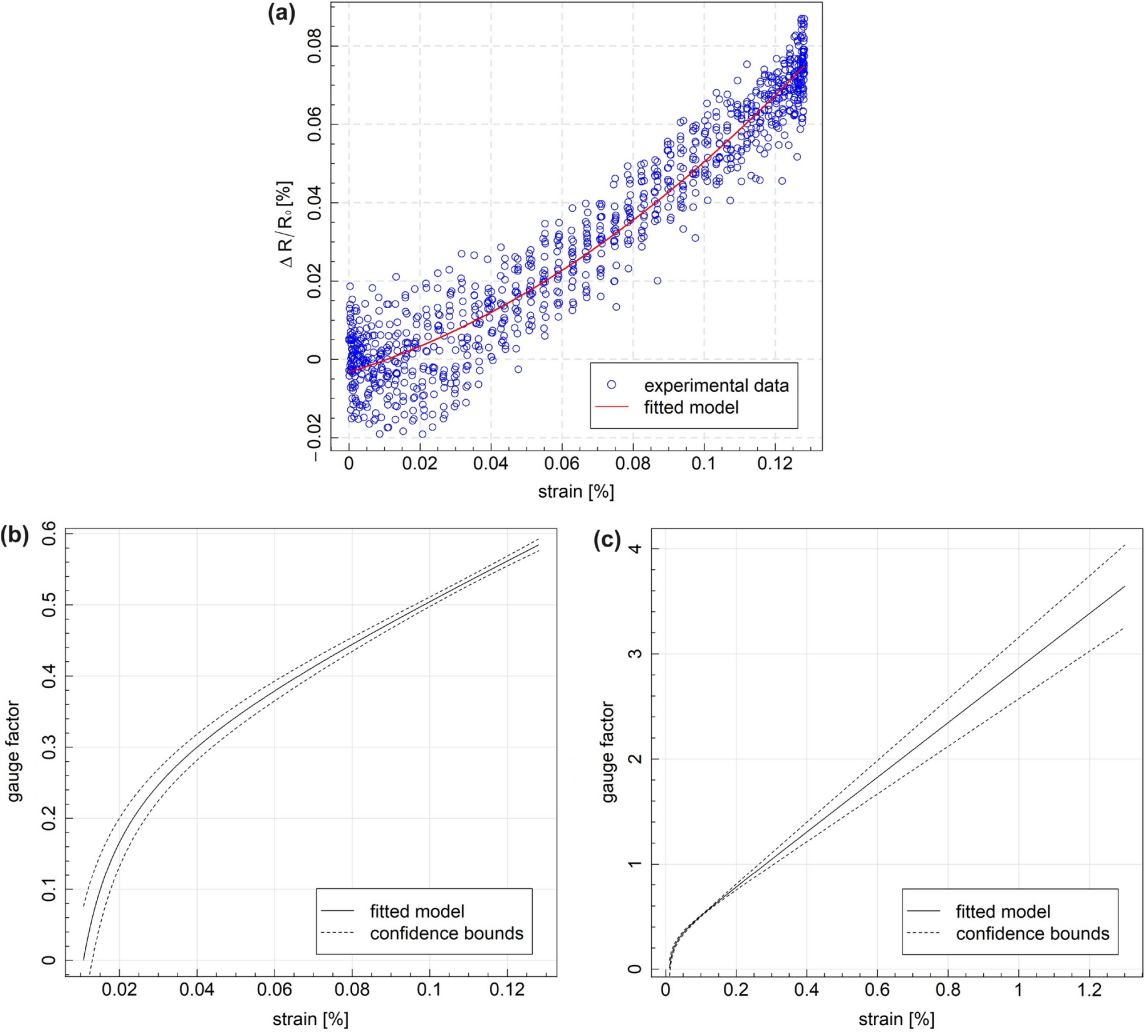
Piezoresistive characteristics of GFRP composites subjected to incremental cyclic loadings: (**a**) the response of fiber no. 3 aligned with the applied force direction with respect to the strain level in this direction, (**b**) gauge factor of the fiber sensor in the experimental strain range and (**c**) gauge factor of the fiber sensor extrapolated from the regression model.

The relationship between $\Delta R/R_0 [\%]$ and the applied strain of the CFRP sample equipped with MXene fiber (FL3) is depicted in Fig. 7a. During cyclic tensile testing, strain gauges recorded a strain value of 0.13% at a stress level of 60 MPa. Analysis of the $\Delta R/R_0 [\%]$-strain curve, derived from regression modeling, indicates that the applied stress range (up to 60 MPa) falls within the linear deformation stage. The $\Delta R/R_0 [\%]$ exhibits a consistent upward trend with increasing strain, even at very low levels (up to 0.13%). Initially, an exponential curve was observed, transitioning into a nearly linear increase beyond 0.04% strain values. Notably, the electrical resistance change rate of MXene fibers displayed minimal fluctuations, demonstrating a strong correlation with stress levels. Table 2 presents estimated quadratic regression model coefficients of the following form:

3 $$\begin{aligned} \Delta R/R_0 [\%] = \beta _0 + \beta _1\varepsilon [\%] + \beta _2\varepsilon [\%]^2 + \epsilon \end{aligned}$$

where $\epsilon$ is the error term, highlighting the robust statistical significance of quadratic term in the response of MXene fiber sensor with respect to the strain $\varepsilon$ (*p*-value < 2e−16).

**Table 2:** Regression model coefficients for FL3 ($\Delta R/R_0 [\%]$).

Coefficient	Estimate	Std. error	*p* value
$\beta _0$	−0.00327	0.00056	8.98e−09
$\beta _1$	0.278	0.023	< 2e−16
$\beta _2$	2.59	0.17	< 2e−16

Adjusted $R^2$: 0.9277, 997 degrees of freedom

The gauge factor (GF) is an algebraic nonlinear function that varies with strain. This calculated equation was used to plot a curve of GF versus strain in Fig. 7b. By importing strain values and model estimated values of $\Delta R/R_0$ into (2), the GF was found to be 0.6 at most, in the range of strain applied in the experiment. While the GF is not the only factor determining calibration, its numerical value is a valuable indicator of the sensor’s sensitivity. The obtained GF can be divided into two linear regions, showing GF values of 0.3 and 0.6 for strain ranges within 0–0.04% and 0.04–0.13%, respectively. A relatively significant resistance change at a minor tensile strain of the MXene fiber sensor is essential in detecting cyclic tensile loading with low mean stress. GF values extrapolated from the regression model for higher strain rate, i.e. in the range comparable with data presented in^31,33,34^, are shown in Fig. 7c.

**Fig. 8:**
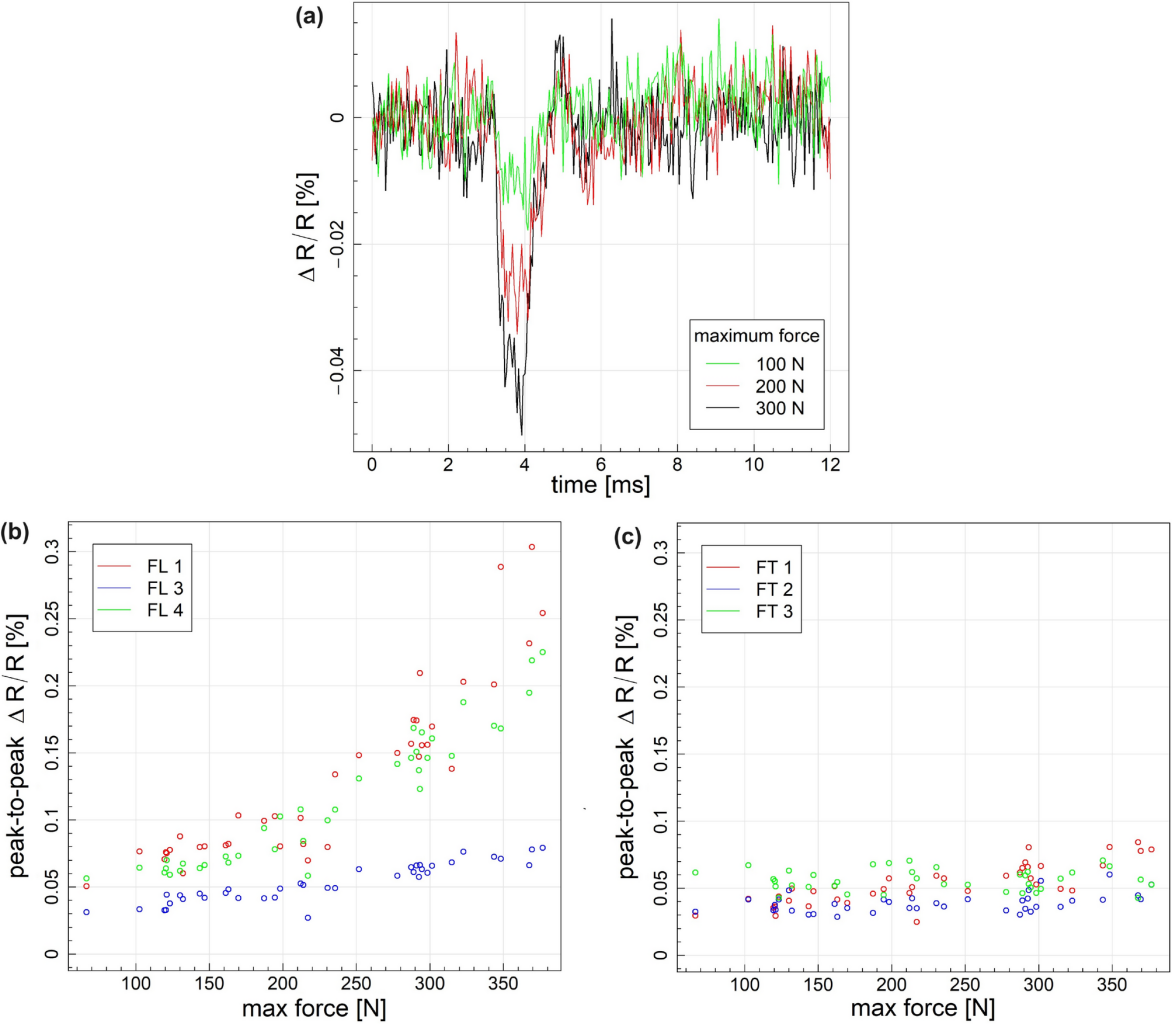
(**a**) Example of signals acquired for fiber no. 3 during tap tests for different maximum force of impacts (**b**) Peak-to-peak fiber signal values with respect to maximum impact force for fibers aligned with the longer edge of the specimen (**c**) Normalized peak-to-peak fiber response with respect to maximum impact force for fibers aligned with the longer edge of the specimen.

To evaluate the sensitivity of the MXene fiber sensor to low-velocity impacts, we conducted a hammer tap test on a CFRP composite material integrated with Mxene fibers. The applied impact load ranged up to 400 N. Concurrently, we recorded the impact force alongside signals from the fibers during the tap test, as depicted in Fig. 8a.

The impact loading led to a decrease in the resistance of the MXene fibers, indicating their tendency to shorten. Notably, the application of the impact force induced localized compression on the upper surface of the composite laminate, where the sensors were positioned. Analysis of the figure revealed that the resistance initially increased and promptly returned to a value reasonably similar to the initial reading. The $\Delta R/R_0$[%] time history exhibited a pattern akin to the impact loading pulse, indicating a proportional relationship between $\Delta R/R_0$[%] and the applied load. Importantly, there was no observable damage on the specimen’s surface, suggesting minimal or no damage incurred. Figure 8b,c presents peak-to-peak signal changes corresponding to the maximum recorded force during the impact for longitudinal and transverse directions. Due to lower bending stress in the transverse direction, for fibers aligned with the shorter edge of the specimen (Fig. 8c), the nominal signal response is significantly lower than for fibers aligned with the longer edge of the specimen due to the higher bending strain (Fig. 8b). For fibers aligned with the shorter edge of the specimen, a positive correlation between the maximum impact force and the electrical resistance change was observed only for FT1 (Fig. 8c), located nearest to the center of the specimen. For fibers aligned with the longer edge of the specimen positive correlation between peak-to-peak values and maximum impact force was observed for all fibers (Fig. 8b). Specifically, the responses of FL1 and FL4 were in very good agreement with the recorded maximum impact load; however, the obtained signal level due to impact was significantly lower for FL3, indicating that the adhesive coating and the fiber-copper port connection limits the strain sensitivity of the fiber. The response of fibers FL1 and FL4 exhibited a non-linear response with respect to maximum impact force, which agrees with findings from cyclic loading tests. There is no statistically significant difference with the response with respect to the impact force between fiber FL1 and FL4.

## Conclusions

In conclusion, this study thoroughly investigates the synthesis, characterization, and application of MXene-based fibers for sensing purposes, particularly in composite materials. Through meticulous experimentation and analysis, several key findings have been established:

First, the synthesis of MXene sheets via selective etching of the Al layer from the Ti_3_AlC_2_ MAX phase has been successfully demonstrated. The morphological properties of MXene sheets, including their transparency and thickness, have been characterized using techniques such as TEM, AFM, and XRD. Additionally, MXene fibers have been produced with a high alignment of MXene sheets along the fiber axis, as revealed by SEM analysis.

Secondly, the electrical properties of MXene fibers have been extensively examined. These fibers exhibit a linear and ohmic response to a range of voltages, indicating their potential as strain sensors. Initial resistance values vary among fibers, with longitudinal fibers showing higher resistance than transverse fibers. Stability tests have shown variations in resistance over time, influenced by factors such as fiber morphology and adhesion.

Thirdly, electromechanical analysis has revealed the piezoresistive characteristics of MXene fiber sensors. A correlation between electrical resistance and applied strain has been established through cyclic tensile tests. The gauge factor of MXene fiber sensors varies with strain, indicating their sensitivity to mechanical deformation.

Lastly, the sensitivity of MXene fiber sensors to low-velocity impacts has been demonstrated through hammer tap tests. Resistance changes in MXene fibers correlate with the applied impact force, suggesting their potential for impact-sensing applications. However, variation in response among fibers has been observed and is influenced by factors such as fiber alignment and adhesive coating.

Overall, this study’s findings highlight the promising potential of MXene-based fibers for various sensing applications, including strain and impact sensing in composite materials. The comprehensive experimental approach and detailed analyses advance our understanding of MXene-based sensors and their integration into structural materials for monitoring and detection purposes.

Further research in this area holds significant promise for the development of advanced sensing technologies with broad applications in engineering, materials science, and beyond. In particular, in future studies, we plan to extend the functionality of our MXene fiber-based system to explore its capabilities in identifying and locating damage within CFRP plates, using more advanced signal processing techniques and further mechanical testing. This will be a meaningful step forward for aerospace SHM applications

## Supplementary Information

Supplementary Information 1.

Supplementary Information 2.

## Data Availability

The datasets used and/or analyzed during the current study available from the corresponding author on reasonable request.
