# Supplementary material for: Enhancing structural health monitoring of fiber-reinforced polymer composites using piezoresistive Ti3C2Tx MXene fibers
Source: Sci Rep. 2025 Jan 19;15:2456. doi: 10.1038/s41598-024-78338-x (PMC11743780; doi:10.1038/s41598-024-78338-x)
Supplement: Supplementary file 1 — Supplementary Information 1. [file 41598_2024_78338_MOESM1_ESM.pdf]

Enhancing Structural Health Monitoring of Fiber-Reinforced Polymer Composites using Piezoresistive Ti3C2TX MXene Fibers

Bircan Haspulat Taymaz[1], Handan Kamis1, Michal Dziendzikowski[2], Kamil Kowalczyk[2],

Krzysztof Dragan[2], and Volkan Eskizeybek[3],*

[1]Department of Chemical Engineering, Faculty of Engineering and Natural Sciences, Konya Technical University, Konya, 42079, Turkiye
[2]Airworthiness Division, Air Force Institute of Technology, Warsaw, 01-494, Poland
[3]Department of Materials Science and Engineering, Faculty of Engineering, Çanakkale Onsekiz Mart University, Çanakkale, 17100, Turkiye
*veskizeybek@comu.edu.tr

Supplementary information

Supplementary video: Fabrication of Mxene fibers using the computer-controlled wet spinning process
